# Impact of FRAXplus adjustments vs FRAX on fracture risk classification in the Swiss Osteoporosis Registry

**DOI:** 10.1093/jbmrpl/ziag120

**Published:** 2026-07-28

**Authors:** Carla Martignoni, Oliver Lehmann, Mathias Wenger, Sven Oser, Martin Toniolo, Gernot Schmid, Ueli Studer, Hans-Rudolf Ziswiler, Christian Steiner, Anna Thoma, Ferdinand Krappel, Piero Pancaldi, Maki Kashiwagi, Diana Frey, HansJörg Häuselmann, Britta Maurer, Stephan Reichenbach, Helena Johansson, Thomas Lehmann, Eugene McCloskey, Judith Everts-Graber

**Affiliations:** Department of Rheumatology and Immunology, Inselspital, Bern University Hospital, University of Bern, Switzerland; Department of Information Technology and Electrical Engineering, ETH Zürich, Zürich, Switzerland; Zentrum für Rheuma- und Knochenerkrankungen, Hirslanden Klinik Im Park, Zürich, Switzerland; Zentrum für Rheuma- und Knochenerkrankungen, Hirslanden Klinik Im Park, Zürich, Switzerland; Zentrum für Rheuma- und Knochenerkrankungen, Hirslanden Klinik Im Park, Zürich, Switzerland; Department of Rheumatology, Lucerne Regional Hospital, Lucerne, Switzerland; OsteoRheuma Bern, Bern, Switzerland; OsteoRheuma Bern, Bern, Switzerland; OsteoRheuma Bern, Bern, Switzerland; Department of Rheumatology and Pain Medicine, Bethesda Hospital, Basel, Switzerland; Department of Orthopedics, European Spine Centre of Excellence, Brig, Switzerland; Rheumatology Outpatient Center, Muralto (Ticino), Switzerland; Gynosense, Women's Health Center, Uster, Switzerland; Gynosense, Women's Health Center, Uster, Switzerland; Zentrum für Rheuma- und Knochenerkrankungen, Hirslanden Klinik Im Park, Zürich, Switzerland; Department of Rheumatology and Immunology, Inselspital, Bern University Hospital, University of Bern, Switzerland; Department of Rheumatology and Immunology, Inselspital, Bern University Hospital, University of Bern, Switzerland; Institute for Social and Preventive Medicine, University of Bern, Switzerland; Mellanby Centre for Musculoskeletal Research, Division of Clinical Medicine, School of Medicine and Population Health, University of Sheffield, Sheffield, United Kingdom; OsteoRheuma Bern, Bern, Switzerland; Mellanby Centre for Musculoskeletal Research, Division of Clinical Medicine, School of Medicine and Population Health, University of Sheffield, Sheffield, United Kingdom; Department of Rheumatology and Immunology, Inselspital, Bern University Hospital, University of Bern, Switzerland; OsteoRheuma Bern, Bern, Switzerland; Department of Diabetes, Endocrinology, Nutritional Medicine and Metabolism, Inselspital, Bern University Hospital, University of Bern, Switzerland

**Keywords:** fracture prediction, FRAX, FRAXplus, osteoporosis, risk factors, BMD

## Abstract

FRAXplus complements the fracture risk assessment tool (FRAX) by incorporating additional risk factors to adjust 10-yr probabilities of major osteoporotic and hip fractures. We aimed to identify which adjustments most strongly influence fracture risk estimates and how they affect risk classification and treatment recommendations in Switzerland. We analyzed data from the Swiss Osteoporosis Registry (2015-2023), which captures all standard FRAX variables plus lumbar spine (LS) BMD, trabecular bone score (TBS), fall history in the previous year, oral glucocorticoid dosages, and the location and recency of prior fractures. Data on hip axis length and duration of type 2 diabetes were not available. A total of 28 708 individuals (88% women; mean age 65.8 ± 11.4 yr) were included, with a median 10-yr probabilities for major osteoporotic fracture (MOF) of 16% (interquartile range [IQR]: 9-24) using standard FRAX and 18% [IQR: 11-28] with FRAXplus adjustments. Most individuals (72%) had higher probabilities with FRAXplus (mean absolute increase: +4.8%), while 27% had lower probabilities (mean absolute decrease: −1.0%). TBS and LS minus FN T-score (LS − FN) influenced nearly all risk estimates, but differences between FRAX and FRAXplus were generally modest. At the individual level, ≥1 fall and recent vertebral or hip fractures produced the largest differences. Applying FRAXplus with Swiss intervention thresholds led to an upward reclassification in 16% of MOF estimates and a downward reclassification in 1%. In summary, FRAXplus adjustments significantly alter fracture risk estimates for most individuals. While LS BMD and TBS affect most probabilities, falls and recent vertebral or hip fractures have the largest absolute impact at the individual level, leading to clinically relevant changes in treatment recommendations according to Swiss guidelines.

## Graphical Abstract

**Graphical Abstract:**
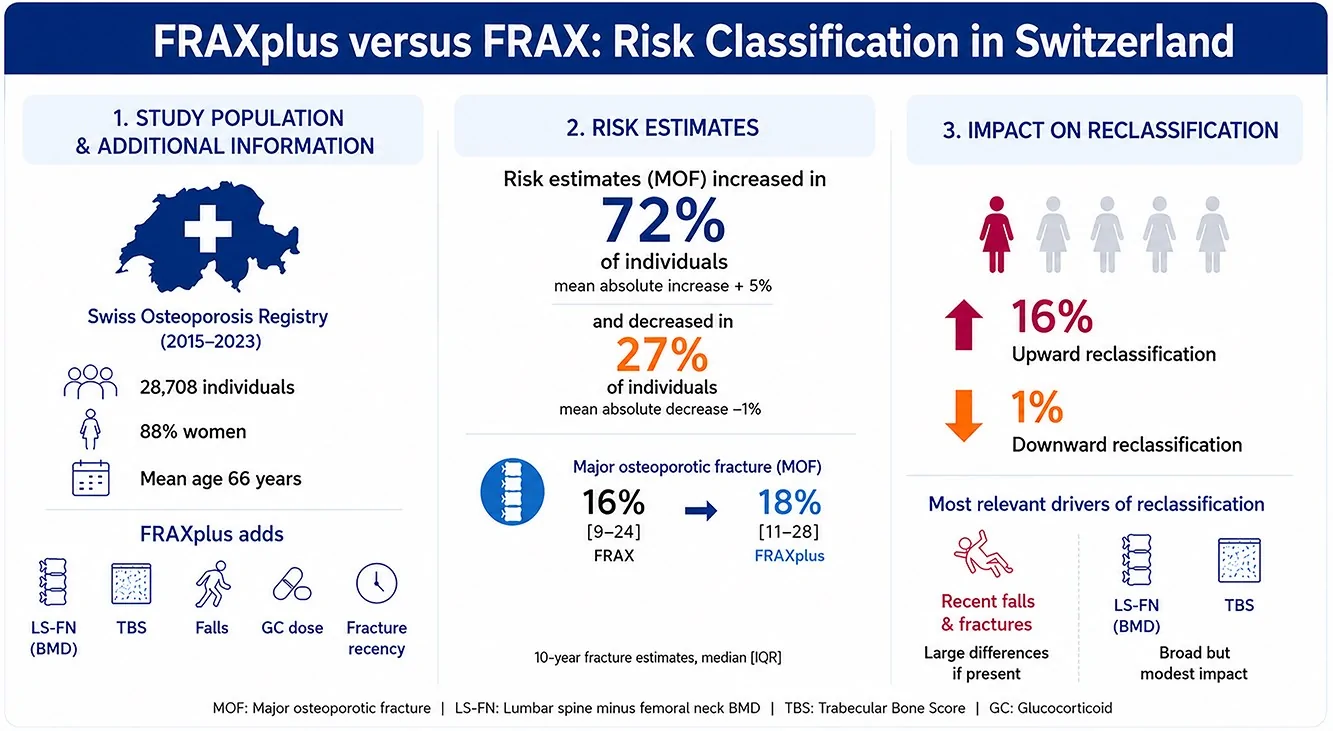

## Introduction

Osteoporosis is characterized by reduced bone mass and increased bone fragility, resulting in a higher risk of fractures. Osteoporotic fractures are associated with substantial morbidity, mortality, and economic burden.^1^ Although osteoporosis was originally defined by the World Health Organization as a BMD T-score ≤ −2.5, most fractures occur in individuals with normal BMD or osteopenia.^2^ Therefore, risk assessment that includes additional clinical risk factors is essential. The most widely used fracture risk assessment tool (FRAX) combines clinical risk factors and optional BMD measurement to estimate the 10-yr probability of major osteoporotic fracture (MOF) (hip, clinical spine, forearm, and humerus) or hip fracture alone.^3^ Specifically, it considers age, sex, BMI, prior fractures (yes/no), parental history of hip fracture, use of oral glucocorticoids, rheumatoid arthritis, smoking, alcohol intake, and femoral neck (FN) BMD.

However, the standard FRAX tool has several limitations, as it does not account for certain clinical factors known to influence fracture risk. To address these gaps, FRAXplus was recently developed as an extension of the classic FRAX model, allowing post hoc adjustments based on additional risk factor information.^4^ These include refinements to existing FRAX variables such as the recency and site of prior fractures^5^^,^^6^ and the dose of oral glucocorticoids,^7^ as well as factors not directly captured by FRAX, such as recent falls,^8^ trabecular bone score (TBS),^9^ lumbar spine (LS) minus FN BMD T-score (LS − FN),^10^ hip axis length,^11^ and duration of type 2 diabetes.^12^ By integrating these parameters, FRAXplus provides a more nuanced and individualized estimate of fracture probability.

We aimed to determine which of the FRAXplus adjustments would be the main drivers of increased (or decreased) fracture risk and how they would affect risk classification, and thereby treatment recommendations in Switzerland.

## Materials and methods

### Study population

This prospective nationwide cohort study was conducted in Switzerland using data from the Swiss Osteoporosis Registry, which has been described previously.^13^^,^^14^ Briefly, this registry was launched in 2015 with the primary aim of evaluating the risk of osteoporotic fractures and to assess treatment effectiveness and safety. Individuals with suspected bone fragility—typically due to previous fractures and/or established risk factors—were consecutively referred for DXA scanning and clinical evaluation, and subsequently enrolled at 12 participating sites across Switzerland, including university and regional hospitals as well as outpatient centers in both urban and rural areas. At baseline, all participants underwent at least 1 DXA scan with a vertebral fracture assessment (VFA), combined with a clinical visit and risk assessment, including tests of fall risk. Standardized data collection comprised demographic variables, risk factors for osteoporotic fractures, BMD T-scores (DXA-derived), TBS, clinical and morphometric fracture history, and details of anti-osteoporotic therapies before and during follow-up. Participants were re-evaluated with DXA and clinical assessments every 2-3 yr depending on their individual fracture risk and therapeutic strategy, with all data collected as part of routine clinical practice. For all calculations, the baseline DXA measurement at registry entry was used.

The study protocol was reviewed and approved by the local ethics committee (KEKBE 2022-01589). All participants provided written informed consent for the further use of their health-related data.

### Fracture assessments

Information on prior fracture history was collected through structured face-to-face interviews and confirmed by medical records from referring general practitioners. Participants also underwent sarcopenia- and fall-related functional tests, including the timed chair stand test. All clinical low-trauma fractures (excluding fingers, toes, and skull) were included as previous fractures in the FRAX input, consistent with FRAX methodology. Morphometric vertebral fractures were systematically assessed using VFA on lateral spine scans (Th6-L4). Images were evaluated according to the semiquantitative method of Genant, and grades 2 and 3 fractures (≥25% reduction in vertebral height) were usually classified as morphometric vertebral fractures^15^; however, final classification was based on clinical judgment and, in some cases, confirmed by CT or MRI. Both clinical and morphometric vertebral fractures were included in the definition of “vertebral fracture” at baseline. If no plausible date could be established (eg, by reviewing prior radiographs), the fracture was assumed to be older than 24 mo (ie, not recent).

### BMD assessments

BMD was measured at the LS (L1-L4), TH, and FN. TBS became available for all patients from January 1, 2014, when the software was implemented. The BMI-adjusted TBS was used in all centers.

### FRAXplus adjustments

For all individuals in the registry, FRAX Switzerland was calculated. Secondary osteoporosis was not included, as it has no effect on FRAX calculations with BMD. Additional risk information beyond the standard FRAX model was incorporated using FRAXplus through univariable adjustments that refined the estimated 10-yr probabilities of major osteoporotic and hip fractures. FRAXplus modifies the original FRAX probabilities using adjustment multipliers derived from published studies, as detailed below). Adjustments for factors already included in FRAX (eg, fracture recency or oral glucocorticoid dose) were only applied when the corresponding FRAX variable was present. In contrast, adjustments for factors not captured by FRAX, such as falls in the preceding year, were applied independently of the FRAX inputs. Each adjustment was applied separately, one factor at a time. When multiple adjustments were applicable for a given individual, the adjustment yielding the highest probability estimate was used. Specifically, adjustments were applied for fracture recency and site, oral glucocorticoid dose, fall history, TBS, and LS-FN. For prior fractures, recency referred to the time since the most recent event (0-1, 2-6, 6-12, or 12-24 mo), and site to 1 of 5 categories (clinical vertebral, humeral, forearm, hip, or other osteoporotic fractures).^6^ Current oral glucocorticoid exposure was categorized as low (<2.5 mg/d), medium (2.5-7.5 mg/d; corresponding to the standard FRAX reference) or high (>7.5 mg/d) according to the average daily prednisolone-equivalent dose, with probabilities adjusted using published age-specific hazard ratios.^7^ Fall history adjustments were based on the number of falls in the previous year (none, 1, 2, or ≥3). TBS-adjusted probabilities were derived using established correction factors, and the effect of LS-FN was modeled with a multiplier of 1.12^LS-–FN^^10^; patients with obvious erroneous LS BMD values were excluded from the dataset. LS-FN captures discordance between skeletal sites and may identify additional fracture risk not reflected by FN BMD alone. Data on hip axis length and diabetes duration were unavailable and therefore not included.

### Risk classification and intervention thresholds

In Switzerland, anti-osteoporotic treatment is recommended based on age-specific risk categories: moderate, high, and very high or imminent fracture risk, as defined by the Swiss Association against Osteoporosis.^16^ Preventive therapy with selective estrogen receptor modulators or oral bisphosphonates is generally considered for individuals with moderate fracture risk, whereas more potent anti-osteoporotic therapies are recommended for those with high fracture risk. The threshold for high fracture risk is defined as at least equivalent to the 10-yr MOF probability of an individual of the same age with a prevalent vertebral fracture, corresponding in Switzerland to ~10% at age 50% and 30% at age 72. Very high or imminent fracture risk is defined as a risk ≥20% higher than the high-risk threshold or following a recent MOF. Patients with very high or imminent fracture risk qualify for anabolic therapy. Since some medication reimbursements in Switzerland rely on a T-score threshold (≤−2.5) or osteoporosis-defining fractures (hip or vertebral fractures), we also compared the intervention thresholds based on FRAXplus with these simpler approaches.

The main “upward driver” was defined as the frequency with which each risk factor accounted for the largest increase in fracture risk among individuals reclassified to a higher risk category. “Impact” was defined as the relative contribution of each risk factor to reclassification relative to its prevalence in the cohort.

### Statistical analysis

Descriptive statistics were employed to summarize baseline characteristics and fracture incidence within the study population. Continuous variables are presented as mean ± SD or median with interquartile range (IQR), depending on the distribution assessed by the Shapiro-Wilk test. Categorical variables are summarized as counts and percentages. Analyses were performed with Python Version 3.9.

## Results

### Baseline characteristics

A total of 28 708 assessments of individuals (mean age 65.8 ± 11.4 yr) were included in the analysis, of whom 87.5% were women. Individuals younger than 40 yr (*n* = 1095) were excluded, while the FRAX age of the 129 individuals aged 90 yr or older was set to 90 yr. The mean BMI was 24.7 ± 4.9 kg/m^2^. A parental history of fracture was reported in 10.6%, current smoking in 9.8% and corticosteroid use in 6.8%, with most users receiving medium or high dosages. Rheumatoid arthritis was present in 3.8% of participants and high alcohol intake (≥3 units/d) was reported in 1.9% (Table 1).

**Table 1:** Baseline cohort characteristics.

Baseline characteristics	*N*	% or Mean ± SD
**Total**	28 708	
**Age (years)**	28 708	65.8 ± 11.4
**Sex** **Female** **Male**	25 120 3588	87.5% 12.5%
**BMI (kg/m** ^**2**^ **)**	28 708	24.7 ± 4.9
**Parental history of hip fracture**	3043	10.6%
**Current smoking (>10 cigarettes per day)**	2813	9.8%
**Corticosteroid use** ^**a**^	1952	6.8%
**Dose of oral glucocorticoids** **None** **Low dosage**^**b**^ **Medium dosage**^**c**^ **High dosage**^**d**^	27 084 222 821 581	94.3% 0.8% 2.9% 2.0%
**Rheumatoid arthritis (confirmed by a rheumatologist)**	1091	3.8%
**Secondary osteoporosis** ^**e**^	N/A	
**High alcohol intake** ^**f**^	545	1.9%
**FN BMD T-score**	28 708	−1.7 ± 0.9
**LS BMD T-score**	28 708	−1.4 ± 1.5
**LS minus FN BMD T-score (LS** − **FN)**	28 708	+0.3 ± 1.3
**TBS** ^**g**^	24 555	1.267 ± 0.13
**Number of self-reported falls in the previous year** **0 falls** **1 fall** **2 falls** **≥3 falls**	27 054 1112 281 261	94.2% 3.9% 1.0% 0.9%
**Previous fracture** ^**g**^	12 345	43.0%
**Previous vertebral fracture** **0-24 mo** **>24 mo**	5631 2299 3332	19.6% 8% 11.6%
**Previous hip fracture** **0-24 mo** **>24 mo**	1528 593 935	5.3% 2.1% 3.3%
**Previous humerus fracture** **0-24 mo** **> 24 mo**	1313 387 926	4.6% 1.3% 3.2%
**Previous forearm fracture** **0-24 mo** **>24 mo**	2813 754 2059	9.8% 2.6% 7.2%
**Previous fracture other location** **0-24 mo** **>24 mo**	5249 2053 3196	18.3% 7.2% 11.1%
**FRAX MOF without BMD**	28 708	19.6 ± 13.5
**FRAX HIP without BMD**	28 708	7.2 ± 9.1
**FRAX MOF with BMD**	28 708	17.8 ± 10.9
**FRAX HIP with BMD**	28 708	5.3 ± 6.7

Abbreviations: FRAX, fracture risk assessment tool; TBS, trabecular bone score; MOF, major osteoporotic fracture.

Dosage of oral glucocorticoid.

^a^<2.5 mg daily of prednisolone or equivalent.

^b^2.5-7.5 mg daily.

^c^>7.5 mg daily.

^d^Secondary osteoporosis was not included, as it does not affect FRAX estimates with BMD.

^e^Alcohol intake 3 or more units/d (self-reported).

^f^Corticosteroid use includes all users, whereas dose categories are limited to patients with available dose data. Totals may therefore differ.

^g^Previous fracture represent individuals, whereas site-specific counts reflect events.

^h^TBS > 1.310 = normal microarchitecture; TBS 1.230-1.310 = partially degraded; TBS < 1.230 = degraded.

The mean FN and LS BMD T-scores were −1.7 ± 0.9 and −1.4 ± 1.5, respectively, corresponding to an average LS-FN of 0.3 ± 1.3. The mean TBS was 1.267 ± 0.13, which corresponds to partially degraded microarchitecture. Falls within the previous year were infrequent, with 94.2% reporting none. Among individuals with falls, 58% also had a history of prior fracture, and 13% of these had sustained a fracture in the previous year. A history of fracture was present in 43% of individuals, most commonly vertebral (19.6%), forearm (9.8%), hip (5.3%), or humeral (4.6%) fractures, the majority (36%) occurring more than 24 mo prior to baseline (Table S1). In total, 16.8% patients were receiving osteoporosis medication, including 11.9% on bisphosphonates and 3.6% on denosumab.

### 10-yr probability of MOF or hip fracture

The median 10-yr MOF probability was 16% [IQR 9-24] with FRAX alone and 18% [IQR 11-28] with FRAXplus adjustments. Overall, 72% of individuals had higher probabilities after adjustment (mean absolute difference + 4.8%), whereas 27% had lower probabilities (mean absolute difference −1.0%).

For hip fracture probability estimates, the median probability with FRAX alone was 3.2% [IQR 1.2-6.8], compared with 3.7% [IQR 1.4-8.0] using FRAXplus adjustments.

**Figure 1:**
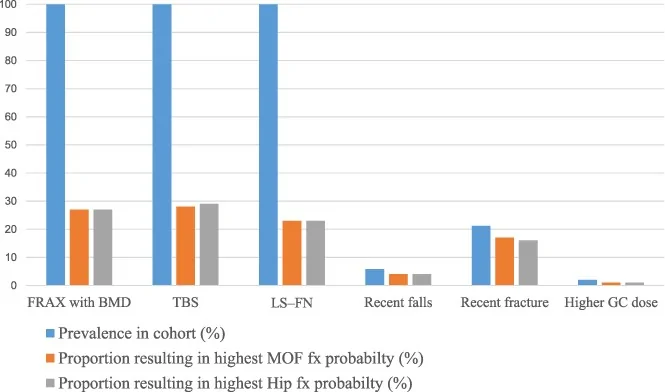
Drivers of highest fracture probability estimates. Prevalence of each FRAXplus adjustment in the cohort (blue/left bar) and proportion of cases in which each adjustment produced the highest 10-yr probability estimate for major osteoporotic fractures (orange/middle bar) or hip fractures (gray/right bar) when BMD was included. Abbreviations: fx, fracture; FRAX, fracture risk assessment tool; TBS, trabecular bone score; LS – FN, LS minus FN BMD T-score; GC, glucocorticoid.

Thus, FRAXplus adjustments modified the 10-yr fracture probabilities for the majority of individuals by incorporating additional risk factor information not included in the original FRAX algorithm. For example, in 73% of all individuals, FRAXplus adjustments produced the highest probability estimate for MOF (including BMD) (Figure 1). In the remaining 27%, the original FRAX calculation remained the source of the maximum probability. Among FRAXplus adjustments that produced the highest probabilities, TBS was the most common contributor (26%), followed by LS-FN (23%). Recent fracture history, falls, and corticosteroid dose contributed smaller proportions. A similar pattern was observed for hip fracture risk: the original FRAX calculation provided the maximum probability in 27% of cases, while FRAXplus adjustments did so in 73%. Similarly to MOF estimates, TBS was the most frequent driver (29%), followed by LS-FN (22%). Taken together, when BMD was included, the highest 10-yr fracture probabilities were most often driven by FRAXplus adjustments for both MOF and hip fracture risk.

**Figure 2:**
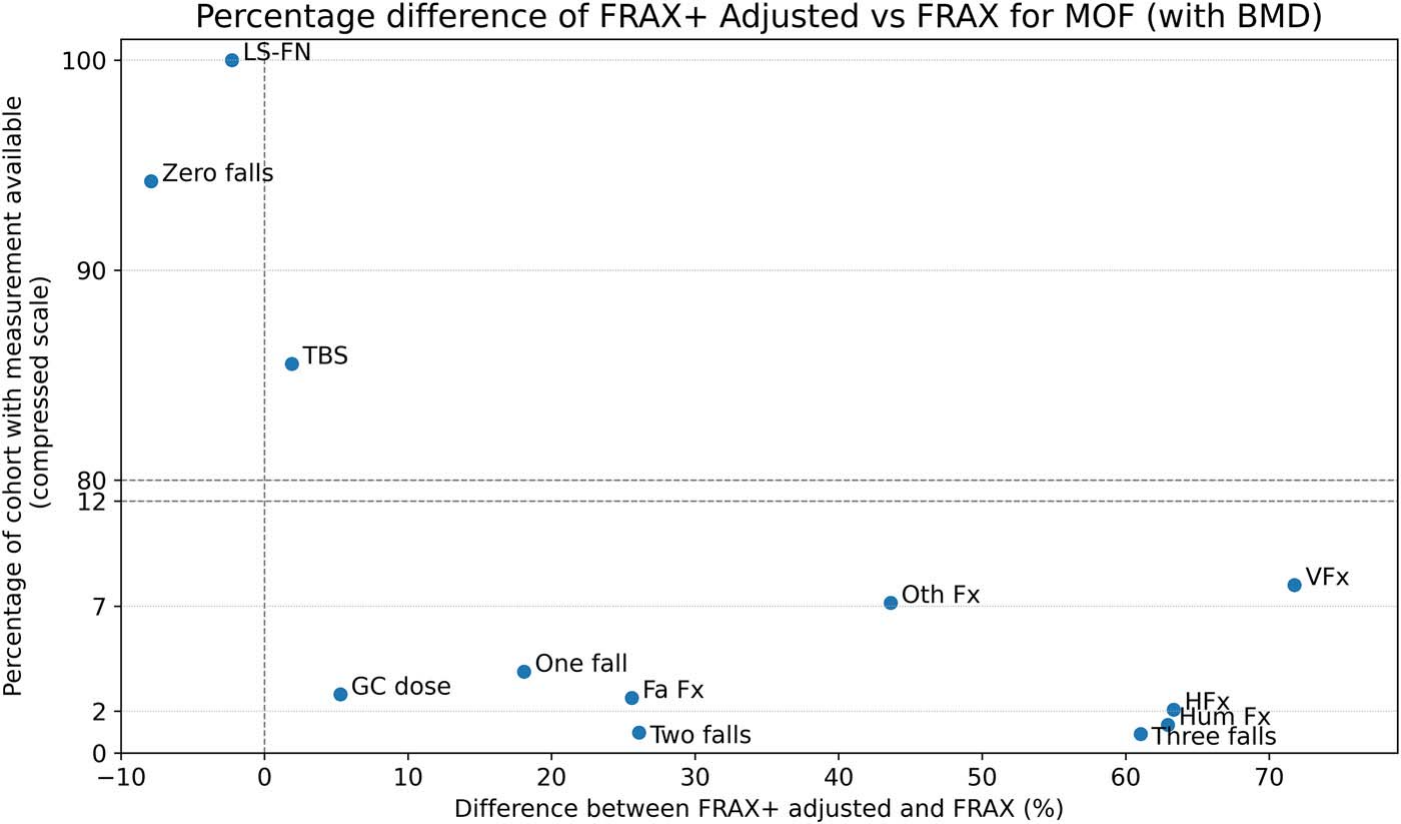
Mean relative percentage difference in 10-yr major osteoporotic fracture probability with FRAXplus adjustments compared with FRAX (with BMD), plotted against the proportion of the cohort with available data for each adjustment. Mean values are calculated among individuals for whom each adjustment was applicable. Abbreviations: FRAX, fracture risk assessment tool; Fx, recent fracture (fa, forearm; oth, other; H, hip; hum, humeral; V, vertebral); GC, glucocorticoid; LS − FN, LS minus FN BMD T-score; TBS. trabecular bone score.

**Figure 3:**
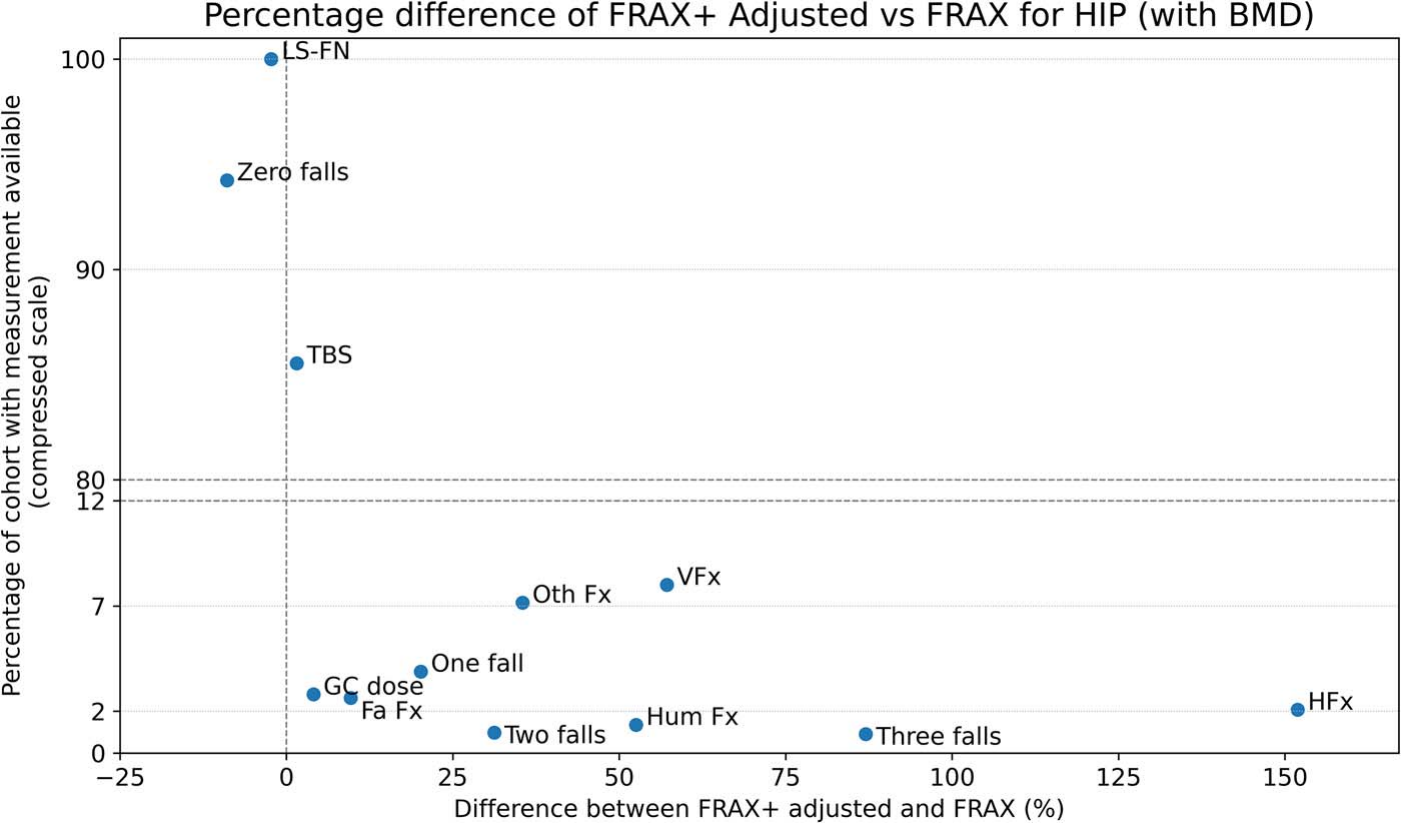
Mean relative percentage difference in 10-yr hip fracture probability with FRAXplus adjustments compared with FRAX (with BMD), plotted against the proportion of the cohort with available data for each adjustment. Mean values are calculated among individuals for whom each adjustment was applicable. Abbreviations: FRAX, fracture risk assessment tool; Fx, recent fracture (fa, forearm; oth, other; H, hip; hum, humeral; V, vertebral); GC, glucocorticoid; LS – FN, LS minus FN BMD T-score; TBS, trabecular bone score.

Figure 2 (MOF estimates) and Figure 3 (hip fracture estimates) show the relative percentage difference following each FRAXplus adjustment compared with FRAX alone, in relation to the proportion of individuals eligible for each adjustment. The strongest upward drivers at the individual level were ≥ 1 fall and recent fractures (particularly vertebral or hip fractures), each producing the largest relative percentage increases when present. In contrast, TBS and LS-FN had frequent but generally modest effects on FRAX probabilities.

### Reclassification analysis

**Figure 4:**
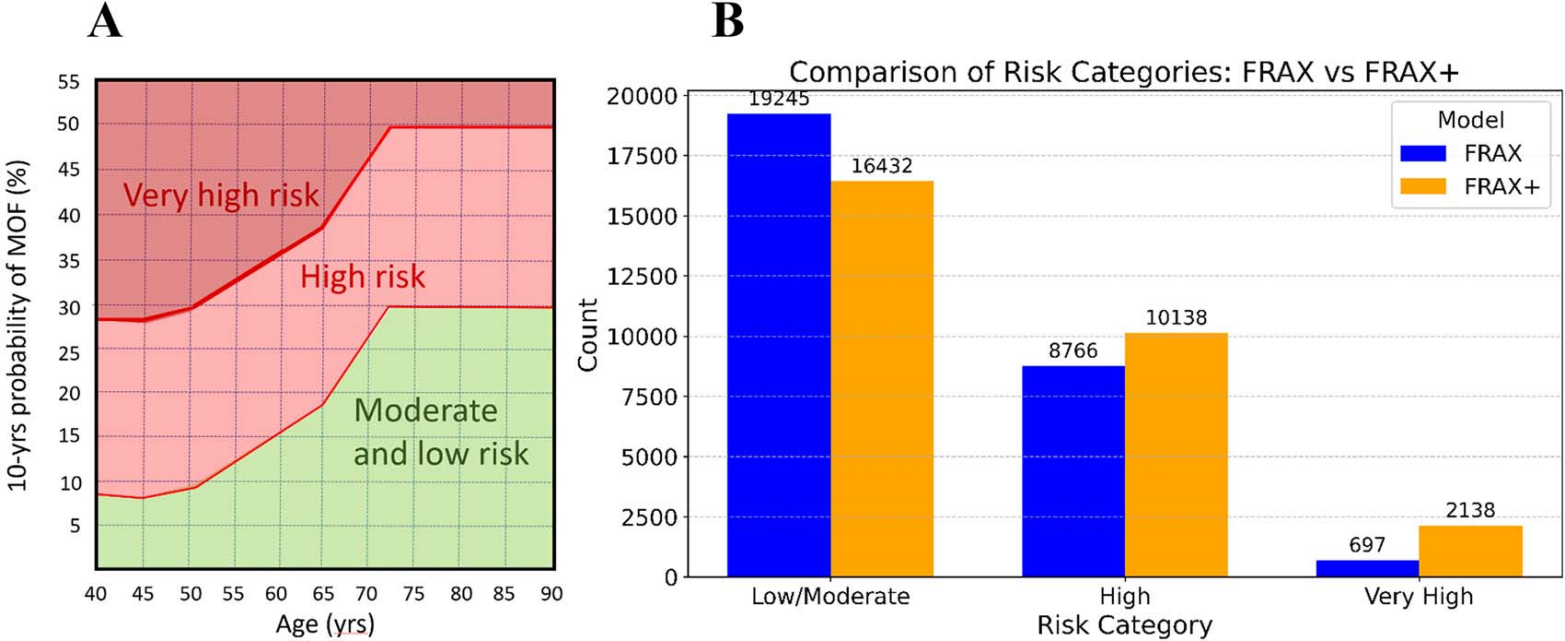
(A) Intervention thresholds for osteoporosis therapy based on 10-yr risk of a major osteoporotic fracture (MOF) in Switzerland, according to Swiss Association against Osteoporosis guidelines from 2020.^16^ (B) Fracture risk classification using FRAX vs FRAXplus adjustments for MOF in the Swiss Osteoporosis Registry.

Applying FRAXplus adjustments together with the Swiss intervention thresholds (Figure 4A) resulted in meaningful shifts in treatment categorization. For MOF, 16% of individuals were reclassified upward (crossing above the intervention threshold) and 1% downward. Specifically, 11% moved from moderate to high risk and 5% from high to very high risk (Figure 4B). Of note, hip fracture probability is not used for risk stratification or treatment thresholds in current Swiss guidelines.^16^

The most relevant drivers for a higher fracture risk category were vertebral fractures, TBS, other fractures, and LS-FN (Table 2). The mean TBS and LS-FN values associated with an upward adjustment of MOF probability were 1.1 ± 0.12 and −1.2 ± 0.65, respectively. However, since TBS and LS-FN adjustments were available for all individuals, we also assessed the relative impact of each adjustment on risk reclassification. This impact reflects how strongly each factor contributed to shifting patients into a different risk category. For example, vertebral fractures accounted for 34% of all upward reclassifications, despite being documented in only 8% of patients. The most influential drivers of upward shifts were fractures and the presence of 3 falls. Downward reclassifications were rare and modest, mainly driven by zero falls, TBS, and LS-FN adjustments.

**Table 2:** Main drivers of risk reclassification in FRAXplus.

Risk factor	Main upward driver	(%)	Impact	Main downward driver	(%)	Impact	Available measurements	(%)
**VFx**	1565	34.1	4.3	0	0	0.0	2299	8
**TBS**	911	19.9	0.2	76	21.2	0.2	24 555	85.5
**Oth Fx**	700	15.3	2.1	0	0	0.0	2053	7.2
**LS–FN**	541	11.8	0.1	80	22.3	0.2	28 708	100
**Hum Fx**	212	4.6	3.5	0	0	0.0	387	1.3
**Hip Fx**	174	3.8	1.8	0	0	0.0	593	2.1
**Forearm Fx**	130	2.8	1.1	0	0	0.0	754	2.6
**Three falls**	130	2.8	3.1	0	0	0.0	261	0.9
**One fall**	113	2.5	0.6	0	0	0.0	1112	3.9
**GC dose**	62	1.4	0.5	0	0	0.0	803	2.8
**Two falls**	49	1.1	1.1	0	0	0.0	281	1.0
**Zero falls**	0	0	0.0	203	56.5	0.6	27 054	94.2
**Total**	4587	100		359	100		28 708	100

Abbreviations: Fx, recent fractures (V, vertebral; Oth, other; Hum, humeral); LS – FN, LS minus FN BMD T-score; GC, glucocorticoid; TBS, trabecular bone score.

Main upward driver: Indicates how often each risk factor was responsible for the largest increase in fracture risk among individuals whose risk category shifted upward. For example, among 4587 patients who moved to a higher risk category, a vertebral fracture was the primary driver of the increase in 1565 cases. Impact: Reflects each risk factor’s contribution to reclassification relative to its prevalence in the For instance, vertebral fractures accounted for 34% of all upward reclassifications, despite being present in only 8% of patients.

Overall, 12 276 patients (43%) were classified as having high or very high fracture risk according to FRAXplus and were therefore above the intervention threshold (Figure 4). In comparison, 13 916 patients (48%) fulfilled at least one of the traditional treatment criteria, namely osteoporosis defined by a FN or LS T-score ≤ −2.5 and/or a prior hip or vertebral fracture. Of these, 10 513 patients (37%) had osteoporosis by BMD, 6660 (23%) had a prior hip and/or vertebral fracture, and 3257 (11%) fulfilled both criteria. Individuals eligible for treatment according to FRAXplus were younger (66 vs 69 yr) and had a greater burden of clinical risk factors than those eligible based on a T-score ≤ −2.5 and/or a prior hip or vertebral fracture **(**Table S2).

## Discussion

This study evaluated the impact of FRAXplus adjustments on fracture risk reclassification compared with traditional FRAX estimates in a large, registry-based Swiss cohort. Specifically, FRAXplus could adjust for prior fracture recency and site,^6^ glucocorticoid dose,^7^ falls in the past year,^8^ TBS^9^ and LS-FN.^10^ In 28 708 individuals, we assessed how these adjustments influenced fracture probabilities, the main drivers of 10-yr MOF and hip fracture estimates, and reclassification according to Swiss intervention threshold. When comparing FRAXplus with the standard FRAX model, adjustments for TBS and LS-FN exerted broad but modest average effects. This likely reflects the fact that these parameters were available for most participants, thereby diluting their overall impact while still contributing meaningfully at the individual level. Use of these adjustments may be most informative in individuals with low LS BMD or TBS or those near treatment thresholds.^17^ The largest increases in fracture probability estimates were observed with ≥1 fall and prior fractures, particularly vertebral fractures for MOF and hip fractures for hip fracture estimates. Interestingly, the absence of a fall in the preceding year slightly reduced the 10-yr fracture probability, which was expected since the population average fall frequency is slightly above zero. This observation is clinically plausible: a person who does not fall is less likely to sustain a fracture, even in the presence of low BMD or other risk factors. Reductions were small, consistent with the purpose of FRAXplus, which is not to lower risk but to refine standard FRAX estimates. Overall, FRAXplus adjustments led to clinically meaningful reclassification of intervention thresholds in Switzerland, with 16% of participants moving to a higher risk category and 1% to a lower one for MOF. One could argue that the strongest drivers of upward reclassification, vertebral and hip fractures, are already clear treatment indications, making FRAXplus less relevant in these cases. Importantly, however, in Switzerland anabolic agents are reimbursed only for patients with a recent MOF and a BMD T-score ≤ −3.5 or a very high fracture risk. Thus, FRAXplus may be particularly useful in patients with prior fractures but higher BMD, where reclassification may influence access to anabolic therapy. When comparing the risk-based classification of FRAXplus, where 43% of patients exceed the intervention threshold, with a simple approach based on a T-score ≤ −2.5 and/or a prior hip or vertebral fracture, the latter would classify 48% of patients as eligible for anti-osteoporotic therapy. These findings demonstrate that FRAXplus-based treatment eligibility does not simply mirror classification based on osteoporosis by BMD or prior hip/vertebral fractures, but identifies a younger and partly different population through incorporation of additional fracture risk factors.

Our findings are consistent with those recently reported in the SUPERB study, which evaluated the effect of FRAXplus adjustments on fracture risk reclassification in 3028 Swedish women aged 75-80 yr.^18^ In that population-based cohort, FRAXplus increased 90% of 10-yr MOF probabilities and reclassified 11% of women from below to above the intervention threshold, improving risk stratification (net reclassification improvement = 4.8%). The predominant drivers of increased probabilities were TBS, hip axis length, falls and recent fractures, comparable to our observations in the Swiss data. These results support improved risk stratification with FRAXplus. Notably, the FRAXplus adjustment factors were derived from 2 external cohorts, which may limit their applicability to our population. However, the main predictors, namely falls and prior MOFs, have been validated in large multinational studies,^8^^,^^19^ suggesting that the FRAXplus modifications are broadly relevant. Nevertheless, the current approach does not account for multivariable relationships or interactions between risk factors, which should be considered in future refinements. In contrast to the SUPERB cohort study, we have not yet analyzed the performance of FRAXplus compared with the standard FRAX model in terms of risk prediction. However, in a previous study, we evaluated traditional and machine learning-based fracture prediction models using the same prospective cohort that included longitudinal fracture data.^14^ Based on feature importance analyses, we found that fracture risk prediction with FRAX can be improved by incorporating additional variables such as LS BMD, history of falls, detailed oral glucocorticoid dosage information, and the recency of prior fractures. These findings suggest potential added value of FRAXplus, which requires formal validation.

### Limitations and strengths

Our study has several limitations. First, we lacked data on hip axis length and the duration of type 2 diabetes, both of which may influence fracture risk and could have affected our estimates. The former, in particular, was an important driver of higher fracture risk estimates in the FRAXplus analysis of the SUPERB cohort.^18^ Second, although data on multiple prior fractures and primary hyperparathyroidism were captured in the registry, the corresponding FRAXplus adjustments had not yet been implemented at the time of the analysis. Third, among individuals with falls or recent fractures, the difference between FRAX and FRAXplus estimates increased markedly, underscoring the importance of carefully documenting fall history and the timing and site of prior fractures. Among 1654 individuals with falls, 58% also had prior fractures and 13% sustained a fracture within the same year, indicating some overlap between risk factors. This overlap may have introduced a minor degree of “double counting” of risk, but it affected only ~13% of individuals who fell and was therefore limited in magnitude. Fourth, some participants were already receiving osteoporosis therapy at baseline, which may have influenced absolute risk estimates. Although previous data suggest that FRAX calibration remains largely stable under treatment,^20^ our recent fracture risk study showed that treatment can have an impact on risk prediction, albeit a small one.^14^ Fifth, the registry includes only at-risk individuals undergoing reimbursed DXA scans, which may limit generalizability. Furthermore, reclassification results are specific to Swiss intervention thresholds.

This study also has notable strengths. The large and clinically representative sample spans a broad spectrum of patients typically seen in osteoporosis clinics, enhancing generalizability. In addition, prior vertebral fractures were radiographically verified using lateral spine scans, allowing the identification of morphometric vertebral fractures. Although this represents a strength of our registry, VFA is not routinely performed in all healthcare systems, potentially reducing the impact of vertebral fracture-based FRAXplus adjustments.

## Conclusion and outlook

In conclusion, FRAXplus alters fracture risk classification compared with the standard FRAX model, increasing most probability estimates. These adjustments shift a relevant number of individuals above treatment thresholds and may help reduce the treatment gap by identifying patients more likely to benefit from antiresorptive or anabolic therapy. A follow-up analysis (2015-2025) will evaluate whether FRAXplus improves fracture prediction compared with FRAX.

## Supplementary Material

Supplementary_Material_FRAXplus_ziag120

## Data Availability

Data can only be made available as de-identified and aggregated results and only upon approval from the Board of the Swiss Osteoporosis Registry Association.
