# Supplementary material for: Impact of FRAXplus adjustments vs FRAX on fracture risk classification in the Swiss Osteoporosis Registry
Source: JBMR Plus. 2026 Jul 28;10(10):ziag120. doi: 10.1093/jbmrpl/ziag120 (PMC13537462; doi:10.1093/jbmrpl/ziag120)
Supplement: Supplementary file 1 [file supplementary_material_fraxplus_ziag120.docx]

| **Fracture** by site and time interval | | |
| --- | --- | --- |
|  | N | % or Mean ± SD |
| Previous vertebral fracture  0–1 months  1–6 months  6–12 months  12–24 months  > 24 months | 5,631  153  600  512  1,034  3,332 | 19.6%  0.5%  2.1%  1.8%  3.6%  11.6% |
| Previous hip fracture  0–1 months  1–6 months  6–12 months  12–24 months  > 24 months | 1,528  16  114  110  353  935 | 5.3%  0.1%  0.4%  0.4%  1.2%  3.3% |
| Previous humerus fracture  0–1 months  1–6 months  6–12 months  12–24 months  > 24 months | 1,313  5  55  74  253  926 | 4.6%  0.0%  0.2%  0.3%  0.9%  3.2% |
| Previous forearm fracture  0–1 months  1–6 months  6–12 months  12–24 months  > 24 months | 2,813  8  128  121  497  2,059 | 9.8%  0.0%  0.4%  0.4%  1.7%  7.2% |
| Previous fracture, other location  0–1 months  1–6 months  6–12 months  12–24 months  > 24 months | 5,249  39  410  360  1,244  3,196 | 18.3%  0.1%  1.4%  1.3%  4.3%  11.1% |

**Supplementary Table 1**: Fracture by site and time interval

|  | **Cohort** | **FRAX+ eligible** | **T-score/fracture eligible** | **Both** | **FRAX+ only** | **T-score/**  **fracture only** | **Neither** |
| --- | --- | --- | --- | --- | --- | --- | --- |
| **N** | **28,708** | **12,276** | **13,916** | **8,815** | **3,461** | **5,101** | **11,331** |
| Age, years | 65.8 ± 11.4 | 66.4 ± 10.7 | 69.0 ± 10.2 | 67.9 ± 10.3 | 62.6 ± 10.8 | 70.9 ± 9.6 | 63.0 ± 12.0 |
| BMI, kg/m² | 24.7 ± 4.9 | 24.2 ± 4.5 | 23.7 ± 4.5 | 23.8 ± 4.4 | 25.2 ± 4.7 | 23.5 ± 4.7 | 25.7 ± 5.3 |
| T-score, femoral neck | -1.7 ± 0.9 | -2.2 ± 0.7 | -2.2 ± 0.7 | -2.4 ± 0.7 | -1.7 ± 0.6 | -1.9 ± 0.8 | -1.2 ± 0.8 |
| T-score, lumbar spine | -1.4 ± 1.5 | -1.9 ± 1.3 | -2.2 ± 1.3 | -2.2 ± 1.3 | -1.2 ± 1.0 | -2.1 ± 1.3 | -0.6 ± 1.4 |
| TBS | 1.3 ± 0.1 | 1.2 ± 0.1 | 1.2 ± 0.1 | 1.2 ± 0.1 | 1.3 ± 0.1 | 1.2 ± 0.1 | 1.3 ± 0.1 |
| FRAX MOF, % (w BMD) | 15.7 [9–24] | 23.1 [16–32] | 21.3 [15–29] | 25.5 [18–34] | 17.9 [13–26] | 15.8 [12–22] | 9.1 [6–14] |
| FRAX+ MOF, % (w BMD) | 18.0 [11–28] | 29.7 [20–38.3] | 25.3 [17–35] | 32.0 [23–41] | 21.4 [16–31] | 17.4 [13–24] | 9.8 [6–15] |
| Female | 25,133 (87.5%) | 11,048 (90.0%) | 12,202 (87.7%) | 7,856 (89.1%) | 3,192 (92.2%) | 4,346 (85.2%) | 9,739 (86.0%) |
| Previous fracture (FRAX) | 12,333 (43.0%) | 9,321 (75.9%) | 8,956 (64.4%) | 7,070 (80.2%) | 2,251 (65.0%) | 1,886 (37.0%) | 1,126 (9.9%) |
| Family history | 3,049 (10.6%) | 2,573 (21.0%) | 1,598 (11.5%) | 1,528 (17.3%) | 1,045 (30.2%) | 70 (1.4%) | 406 (3.6%) |
| Smoking | 2,820 (9.8%) | 1,405 (11.4%) | 1,299 (9.3%) | 1,009 (11.4%) | 396 (11.4%) | 290 (5.7%) | 1,125 (9.9%) |
| Glucocorticoids | 1,963 (6.8%) | 1,227 (10.0%) | 748 (5.4%) | 657 (7.5%) | 570 (16.5%) | 91 (1.8%) | 645 (5.7%) |
| Rheumatoid arthritis | 1,077 (3.8%) | 657 (5.4%) | 399 (2.9%) | 344 (3.9%) | 313 (9.0%) | 55 (1.1%) | 365 (3.2%) |
| High alcohol intake | 558 (1.9%) | 309 (2.5%) | 271 (1.9%) | 218 (2.5%) | 91 (2.6%) | 53 (1.0%) | 196 (1.7%) |
| Prior hip fracture | 1,528 (5.3%) | 1,215 (9.9%) | 1,528 (11.0%) | 1,215 (13.8%) | 0 (0.0%) | 313 (6.1%) | 0 (0.0%) |
| Prior vertebral fracture | 5,631 (19.6%) | 4,187 (34.1%) | 5,631 (40.5%) | 4,187 (47.5%) | 0 (0.0%) | 1,444 (28.3%) | 0 (0.0%) |

**Suppl. Table 2**: Characteristics of individuals eligible for treatment according to FRAXplus versus T-score/fracture criteria (T-score ≤ –2.5 and/or prior vertebral or hip fracture
